# Constitutive Equations for Analyzing Stress Relaxation and Creep of Viscoelastic Materials Based on Standard Linear Solid Model Derived with Finite Loading Rate

**DOI:** 10.3390/polym14102124

**Published:** 2022-05-23

**Authors:** Che-Yu Lin, Yi-Cheng Chen, Chen-Hsin Lin, Ke-Vin Chang

**Affiliations:** 1 Institute of Applied Mechanics, College of Engineering, National Taiwan University, No. 1, Sec. 4, Roosevelt Road, Taipei 10617, Taiwan; david1998chen@gmail.com; 2 Department of Mechanical Engineering, College of Engineering, National Taiwan University, No. 1, Sec. 4, Roosevelt Road, Taipei 10617, Taiwan; estherlin2000918@gmail.com; 3 Department of Physical Medicine and Rehabilitation and Community and Geriatric Research Center, National Taiwan University Hospital Bei-Hu Branch, Taipei 10845, Taiwan; 4 Department of Physical Medicine and Rehabilitation, National Taiwan University College of Medicine, Taipei 10048, Taiwan; 5 Center for Regional Anesthesia and Pain Medicine, Wang-Fang Hospital, Taipei Medical University, Taipei 11600, Taiwan

**Keywords:** viscoelasticity, viscoelastic models, viscoelastic properties, mechanical properties, material characterization, finite element, modeling and simulation, computational materials science

## Abstract

The viscoelastic properties of materials such as polymers can be quantitatively evaluated by measuring and analyzing the viscoelastic behaviors such as stress relaxation and creep. The standard linear solid model is a classical and commonly used mathematical model for analyzing stress relaxation and creep behaviors. Traditionally, the constitutive equations for analyzing stress relaxation and creep behaviors based on the standard linear solid model are derived using the assumption that the loading is a step function, implying that the loading rate used in the loading process of stress relaxation and creep tests is infinite. Using such constitutive equations may cause significant errors in analyses since the loading rate must be finite (no matter how fast it is) in a real stress relaxation or creep experiment. The purpose of this paper is to introduce the constitutive equations for analyzing stress relaxation and creep behaviors based on the standard linear solid model derived with a finite loading rate. The finite element computational simulation results demonstrate that the constitutive equations derived with a finite loading rate can produce accurate results in the evaluation of all viscoelastic parameters regardless of the loading rate in most cases. It is recommended that the constitutive equations derived with a finite loading rate should replace the traditional ones derived with an infinite loading rate to analyze stress relaxation and creep behaviors for quantitatively evaluating the viscoelastic properties of materials.

## 1. Introduction

Polymers are fundamental materials in numerous industries such as aerospace, automobile, biomedicine, cosmetics, electronics, packaging, sports, textile, rubber and plastics, and so on. The applications of polymers are still advancing and increasing rapidly, thanks to their exceptional mechanical properties. The mechanical behaviors of all polymers are viscoelastic, meaning that they exhibit time-dependent mechanical behaviors of both viscous fluids and elastic solids [1,2,3,4,5]. By measuring and analyzing the viscoelastic behaviors (such as stress relaxation and creep) of a polymer when it is subjected to loading, its viscoelastic properties can be quantitatively evaluated [6,7,8,9,10]. The viscoelastic properties of polymers have profound impacts on applications since they not only determine the performance and quality of products in relevant industries but also determine the ease and success of manufacturing processes during production [1]. The viscoelastic properties can also be applied to classify and compare different types of polymers [11,12,13,14]. In addition to polymer engineering and science, viscoelastic properties have important applications in many other fields. For example, the viscoelastic properties can be served as quantities to differentiate normal and pathological tissues in clinical diagnosis [7,15,16,17,18,19,20,21], to evaluate the functional statuses of engineered tissues in tissue engineering [22,23,24], to understand the conditions of cells and the disease evolutions in biological and medical sciences [25,26], and so on. For successful applications of viscoelastic properties, it is an important issue regarding how to accurately evaluate them. In order to evaluate the viscoelastic properties of materials accurately, viscoelastic mathematical models and the accurate presentations of their solutions are required.

**Figure 1:**
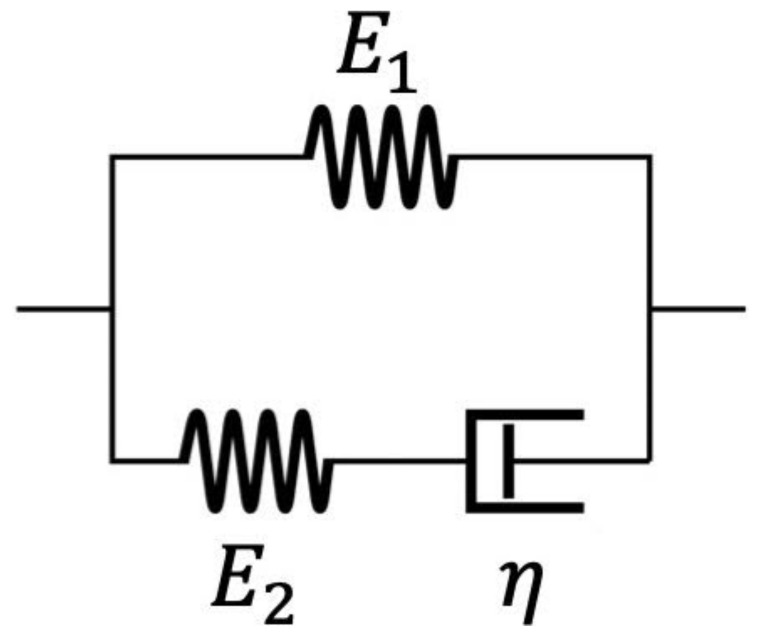
The Maxwell form of the standard linear solid model. $E_{1}$, $E_{2}$ and η are three parameters in the model relevant to the viscoelastic properties.

The Maxwell form of the standard linear solid model (abbreviated as the standard linear solid model in the following text), as shown in Figure 1, is a classical viscoelastic mathematical model commonly applied to analyze experimentally measured or computationally simulated viscoelastic behaviors such as stress relaxation and creep for evaluating the viscoelastic properties of materials [27,28,29,30]. Other than the standard linear solid model, there are many other types of viscoelastic models available to use, including more complicated models such as generalized Maxwell and Kelvin models [31,32,33,34,35,36,37,38,39,40,41,42,43,44] and fractional order models [11,45,46,47]. Nevertheless, the standard linear solid model is still one of the most common and popular viscoelastic models because of its simplicity and high applicability. The standard linear solid model has been widely applied to analyze the viscoelastic behaviors of a great variety of materials, including polymers [48,49,50,51,52,53,54,55,56,57,58,59,60], single cells [61,62,63,64,65], blood vessels [66,67], heart muscles [68], cartilages [69,70], intervertebral discs [71,72], hydrogels [55,73,74,75], and various biomaterials, to name a few.

In literature, traditionally, the stress relaxation behavior based on the standard linear solid model is described by the following equation [30,76,77]:

(1) $$\sigma_{relaxation}\left(t\right)=\left(E_{1}+E_{2}e^{\frac{-t}{\tau_{R}}}\right)\varepsilon_{0}$$

where t is time, $\sigma_{relaxation}\left(t\right)$ is the stress as a function of time during the stress relaxation process, $\varepsilon_{0}$ is the constant strain during the stress relaxation process, $\tau_{R}=\eta /E_{2}$ is the relaxation time constant, $E_{1}$, $E_{2}$ and η are three parameters in the standard linear solid model relevant to the viscoelastic properties. On the other hand, traditionally, the creep behavior based on the standard linear solid model is described by the following equation [30,76,77]:

(2) $$\varepsilon_{creep}\left(t\right)=\frac{\sigma_{0}}{E_{1}}\left(1-\frac{E_{2}}{E_{1}+E_{2}}e^{\frac{-t}{\tau_{C}}}\right)$$

where $\varepsilon_{creep}\left(t\right)$ is the strain as a function of time during the creep process, $\sigma_{0}$ is the constant stress during the creep process, $\tau_{C}=\eta \left(E_{1}+E_{2}\right)/E_{1}E_{2}$ is the creep time constant (or called the retardation time constant).

These two equations are widely applied to analyze experimentally measured or computationally simulated stress relaxation and creep behaviors for evaluating the viscoelastic properties of materials. However, these two equations are derived based on the assumption that the loading is a step function, implying that the rate of loading is infinite (i.e., extremely fast). Using such equations may cause significant errors in analyses since the rate of loading (no matter how fast it is) must be finite in a real stress relaxation or creep experiment. Some previous experimental studies have reported that the patterns of viscoelastic behaviors depend on the loading rate, demonstrating the need for the constitutive equations that take the finite loading rate into account to analyze the strain-rate-dependent viscoelastic behaviors [78,79,80,81].

The purpose of this paper is to introduce the constitutive equations for analyzing stress relaxation and creep behaviors based on the standard linear solid model derived with a finite loading rate. Finite element computational simulation will be conducted to compare the accuracy of the constitutive equations derived with finite loading rate to that of the traditional equations derived with infinite loading rate. The results will demonstrate that, compared to the traditional constitutive equations derived with infinite loading rate, the constitutive equations derived with finite loading rate can produce more accurate results in the evaluation of all viscoelastic parameters regardless of the loading rate in most cases. One of the main intentions of this paper is to provide practical tools for the readers to directly analyze their data. Therefore, we provide the MATLAB (R2021a; Mathworks, Natick, MA, USA) computer programming codes for analyzing (i.e., curvefitting) stress relaxation and creep data based on the constitutive equations introduced in this paper. Only the most important mathematical equations are mentioned in the main text of the paper; however, the readers can find the detailed derivation of each equation that appeared in the paper in Supplementary Materials File S1.

## 2. Materials and Methods

### 2.1. Overview

The constitutive equations (both our proposed ones derived with finite loading rate and the traditional ones derived with infinite loading rate) for analyzing stress relaxation and creep behaviors based on the standard linear solid model will be introduced in Section 2.2 and Section 2.3, respectively. Intuitively, it seems that stress relaxation and creep are two different viscoelastic behaviors. However, actually, stress relaxation and creep behaviors are interrelated and can be regarded as the two sides of the same coin [82,83,84]. In the stress relaxation test, we control the strain (by controlling the strain rate) in the loading process and then monitor how the stress changes with time during the stress relaxation process. On the other hand, in the creep test, we control the stress (by controlling the stress rate) in the loading process and then monitor how the strain changes with time during the creep process. Since the processes in the stress relaxation and creep tests are very similar, the content in Section 2.2 for introducing the constitutive equations for stress relaxation and the content in Section 2.3 for introducing the constitutive equations for creep will be very similar. However similar these two sections are, the readability could be higher if these two sections could be presented as two separate sections and written independently. Therefore, Section 2.2 and Section 2.3 can be viewed as two independent sections. Please note the differences when reading these two sections: the main difference between these two sections is that the term “stress” in Section 2.2 will be replaced by “strain” in Section 2.3, the term “strain” in Section 2.2 will be replaced by “stress” in Section 2.3, while the term “stress relaxation” in Section 2.2 will be replaced by “creep” in Section 2.3.

Section 2.4 will describe the details of using finite element computational simulation to compare the accuracy of our proposed constitutive equations derived with finite loading rate to that of the traditional constitutive equations derived with infinite loading rate. Finite element computational simulation will be used to simulate a series of stress relaxation and creep curves tested with different loading rates (from extremely slow to extremely fast). Each of the simulated stress relaxation and creep curves will be curvefitted by the two equation forms, respectively. In order to determine the accuracy of each equation form, the viscoelastic properties designated in the finite element simulation software suite during the simulation (served as the true viscoelastic properties of materials) will be compared to those obtained by each of the two equation forms.

### 2.2. Two Constitutive Equation Forms for Analyzing Stress Relaxation Behavior Based on the Standard Linear Solid Model

#### 2.2.1. Introduction to the Stress Relaxation Test

**Figure 2:**
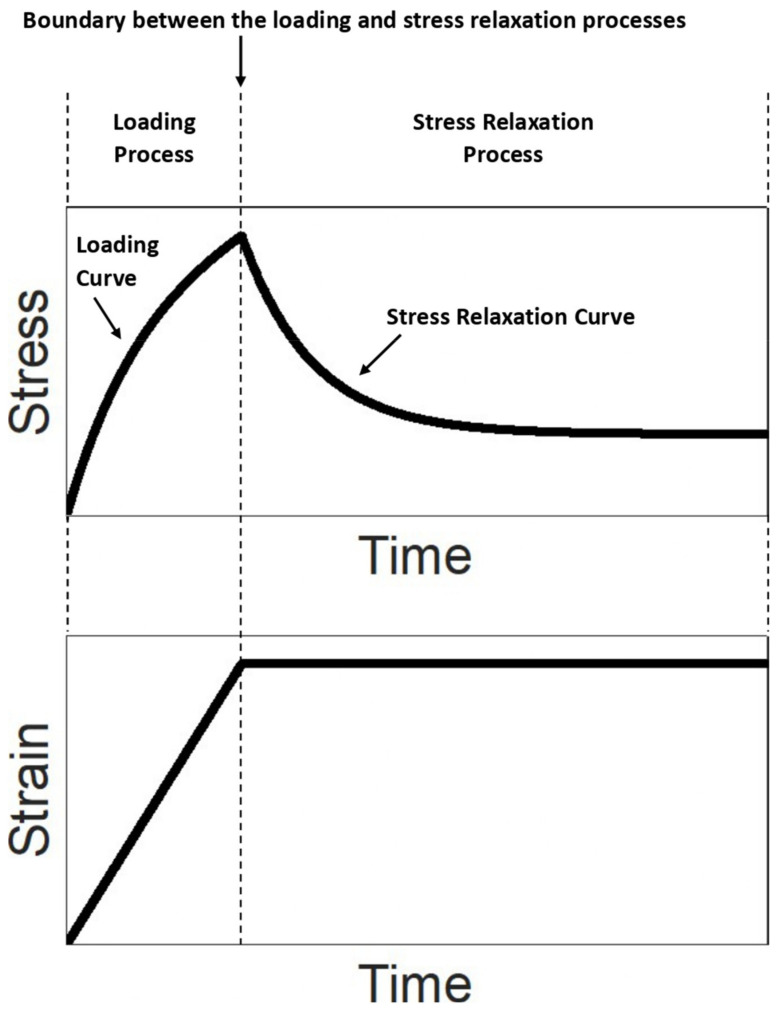
Illustration of the stress–time and strain–time relationships in the stress relaxation test.

There are two sequential processes (the term “process” here can be interpreted as the action performed on the material in the test) in the stress relaxation test: loading process and stress relaxation process (Figure 2).

In the loading process, an external load is applied to the material with a constant strain rate from the initial state (the state in which the material has no stress and strain) until a prescribed strain value is reached. In the loading process, the stress of the material increases monotonically with time; therefore, the stress–time curve (called the loading curve) is a monotonically increasing curve (Figure 2). The strain of the material increases linearly with time since the strain rate is constant.

The solution for describing the stress–time curve of the loading process in the stress relaxation test based on the standard linear solid model is:

(3) $$\sigma_{loading}\left(t\right)=E_{1}rt+E_{1}r\left(\tau_{C}-\tau_{R}\right)\left(1-e^{\frac{-t}{\tau_{R}}}\right)$$

Please refer to Supplementary Materials File S1 for the derivation of Equation (3).

Once the prescribed strain value is reached at the end of the loading process, the strain is held as constant at that prescribed strain for a period of time to trigger the stress relaxation behavior. In the stress relaxation process, during the period when the strain is constant, the stress decreases monotonically with time until constant stress is reached (Figure 2). The stress–time curve of the stress relaxation process is called the stress relaxation curve.

The solution for describing the stress–time curve of the stress relaxation process in the stress relaxation test based on the standard linear solid model is:

(4) $$\sigma_{relaxation}\left(t\right)=\left(\sigma_{0}-E_{1}\varepsilon_{0}\right)e^{\frac{-t}{\tau_{R}}}+E_{1}\varepsilon_{0}$$

Please refer to Supplementary Materials File S1 for the derivation of Equation (4).

Once the stress relaxation test is completed, a loading curve (from the loading process) and a stress relaxation curve (from the stress relaxation process) can be obtained (Figure 2). By using the solution for describing the stress relaxation behavior to analyze (i.e., curvefit) the stress relaxation curve, the corresponding viscoelastic properties of the material can be evaluated. The accuracy of the evaluation depends on both the strain rate used in the loading process and the constitutive equation form chosen for the analysis. Depending on considering the strain rate as finite or infinite, there are two constitutive equation forms based on the standard linear solid model for curvefitting the stress relaxation curve, introduced as follows.

#### 2.2.2. Constitutive Equation Derived with “Finite” Loading Rate for Analyzing Stress Relaxation Behavior

The constitutive equation derived with finite loading rate for curvefitting stress relaxation curve is derived based on the fact that the strain rate used in the loading process in the stress relaxation test is a finite constant. This constitutive equation describes a realistic situation since the strain rate must be a finite constant (no matter how slow or fast it is) in a real stress relaxation test.

The solution for describing the stress–time relationship of the loading process, i.e., Equation (3), and the solution for describing the stress–time relationship of the stress relaxation process, i.e., Equation (4), are used in the derivation of the constitutive equation considering the strain rate as finite for curvefitting stress relaxation curve.

Letting $t=t^{\prime }$ (the time point at the end of the loading process, which is also the time point at the beginning of the stress relaxation process) in Equation (3), we can obtain $\sigma_{0}$ as a function of $\varepsilon_{0}$:

(5) $$\begin{array}{ll} \sigma_{loading}\left(t^{\prime }\right)=\sigma_{0} & =E_{1}rt^{\prime }+E_{1}r\left(\tau_{C}-\tau_{R}\right)\left(1-e^{\frac{-t^{\prime }}{\tau_{R}}}\right)\\ & =E_{1}\varepsilon_{0}+E_{1}r\left(\tau_{C}-\tau_{R}\right)\left(1-e^{\frac{-\varepsilon_{0}}{\tau_{R}\cdot r}}\right) \end{array}$$

In Equation (5), we have used the relationship $\varepsilon_{0}=rt^{\prime }$, meaning that the strain at the end of the loading process is equal to the strain rate multiplies the time duration of the loading process. Substituting Equation (5) into Equation (4), the stress–time relationship of the stress relaxation process can be expressed as:

(6) $$\sigma_{relaxation}\left(t\right)=\left[E_{1}r\left(\tau_{C}-\tau_{R}\right)\left(1-e^{\frac{-\varepsilon_{0}}{\tau_{R}\cdot r}}\right)\right]e^{\frac{-t}{\tau_{R}}}+E_{1}\varepsilon_{0}$$

If the time-dependent ordinary exponential function in Equation (6), i.e., $e^{-t/\tau_{R}},$ is replaced by a stretched-exponential function $e^{-{\left(t/\tau_{R}\right)}^{k}}$, the curvefitting ability of the constitutive equation can be greatly improved so that the constitutive equation can account for the stress relaxation behaviors of most of the materials. By doing so, Equation (6) becomes:

(7) $$\sigma_{relaxation}\left(t\right)=\left[E_{1}r\left(\tau_{C}-\tau_{R}\right)\left(1-e^{\frac{-\varepsilon_{0}}{\tau_{R}\cdot r}}\right)\right]e^{-{\left(\frac{t}{\tau_{R}}\right)}^{k}}+E_{1}\varepsilon_{0}$$

where k∈ℝ and 0<k≤1. Equation (7) is namely the constitutive equation derived with finite strain rate for curvefitting stress relaxation curve based on the standard linear solid model. In Equation (7), $E_{1}$, $E_{2}$ and η are three parameters in the standard linear solid model relevant to the viscoelastic properties, and they are unknown variables to be determined in the analysis. The values of r (i.e., the strain rate used in the loading process) and $\varepsilon_{0}$ (i.e., the constant strain during the stress relaxation process) are known since they are preset parameters designated by the tester in the stress relaxation test. r and $\varepsilon_{0}$ can also be obtained from the data of the test.

The practical method of using Equation (7) to analyze stress relaxation behavior to obtain the viscoelastic properties of materials is: Using Equation (7) to curvefit the stress relaxation curve, the values of $E_{1}$, $\tau_{C}$ and $\tau_{R}$ can be obtained. By solving two simultaneous equations $\tau_{C}=\eta \left(E_{1}+E_{2}\right)/E_{1}E_{2}$ and $\tau_{R}=\eta /E_{2}$, the values of $E_{2}$ and η can be obtained. Therefore, all of the three parameters in the standard linear solid model relevant to the viscoelastic properties, $E_{1}$, $E_{2}$ and η, can be obtained. Please see Supplementary Materials File S2 for a MATLAB computer programming code for curvefitting stress relaxation curve based on using Equation (7).

#### 2.2.3. Constitutive Equation Derived with “Infinite” Loading Rate for Analyzing Stress Relaxation Behavior

The constitutive equation derived with infinite loading rate for curvefitting stress relaxation curve is derived based on the assumption that the strain rate used in the loading process approaches infinity (i.e., extremely fast):

(1) $$\sigma_{relaxation}\left(t\right)=\left(E_{1}+E_{2}e^{\frac{-t}{\tau_{R}}}\right)\varepsilon_{0}$$

Please refer to Supplementary Materials File S1 for the derivation of Equation (1).

The practical method of using Equation (1) to analyze stress relaxation behavior to obtain the viscoelastic properties of materials is: Using Equation (1) to curvefit the stress relaxation curve, the values of $E_{1}$, $E_{2}$ and $\tau_{R}$ can be obtained. Since $E_{2}$ and $\tau_{R}$ have been obtained, and knowing that $\tau_{R}=\eta /E_{2}$, the value of η can be obtained. Therefore, all of the three parameters in the standard linear solid model relevant to the viscoelastic properties, $E_{1}$, $E_{2}$ and η, can be obtained. Please see Supplementary Materials File S2 for a MATLAB computer programming code for curvefitting stress relaxation curve based on using Equation (1).

### 2.3. Two Constitutive Equation Forms for Analyzing Creep Behavior Based on the Standard Linear Solid Model

#### 2.3.1. Introduction to the Creep Test

**Figure 3:**
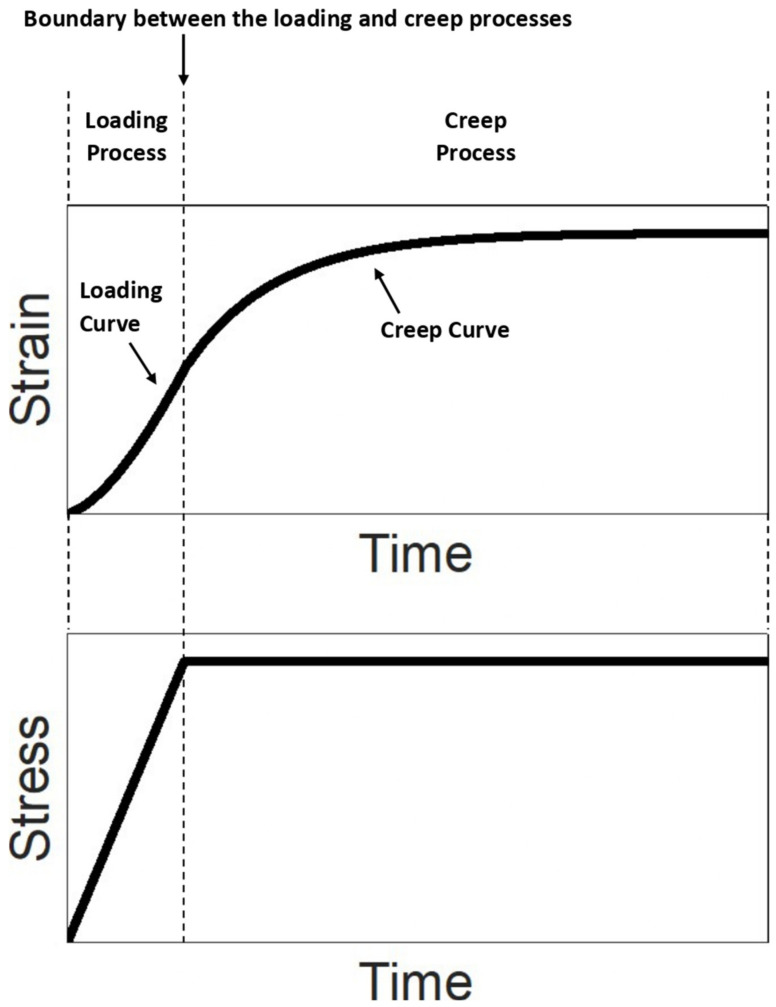
Illustration of the strain–time and stress–time relationships in the creep test.

There are two sequential processes (the term “process” here can be interpreted as the action performed on the material in the test) in the creep test: loading process and creep process (Figure 3).

In the loading process, an external load is applied to the material with a constant stress rate from the initial state (the state in which the material has no stress and strain) until a prescribed stress value is reached. In the loading process, the strain of the material increases monotonically with time; therefore, the strain–time curve (called the loading curve) is a monotonically increasing curve (Figure 3). The stress of the material increases linearly with time since the stress rate is constant.

The solution for describing the strain–time curve of the loading process in the creep test based on the standard linear solid model is:

(8) $$\varepsilon_{loading}\left(t\right)=\frac{r}{E_{1}}t+\frac{r}{E_{1}}\left(\tau_{R}-\tau_{C}\right)\left(1-e^{\frac{-t}{\tau_{C}}}\right)$$

Please refer to Supplementary Materials File S1 for the derivation of Equation (8).

Once the prescribed stress value is reached at the end of the loading process, the stress is held as constant at that prescribed stress for a period of time to trigger the creep behavior. In the creep process, during the period when the stress is constant, the strain increases monotonically with time until a constant strain is reached (Figure 3). The strain–time curve of the creep process is called the creep curve.

The solution for describing the strain–time curve of the creep process in the creep test based on the standard linear solid model is:

(9) $$\varepsilon_{creep}\left(t\right)=\left(\varepsilon_{0}-\frac{\sigma_{0}}{E_{1}}\right)e^{\frac{-t}{\tau_{C}}}+\frac{\sigma_{0}}{E_{1}}$$

Please refer to Supplementary Materials File S1 for the derivation of Equation (9).

Once the creep test is completed, a loading curve (from the loading process) and a creep curve (from the creep process) can be obtained (Figure 3). By using the solution for describing the creep behavior to analyze (i.e., curvefit) the creep curve, the corresponding viscoelastic properties of the material can be evaluated. The accuracy of the evaluation depends on both the stress rate used in the loading process and the constitutive equation form chosen for the analysis. Depending on considering the stress rate as finite or infinite, there are two constitutive equation forms based on the standard linear solid model for curvefitting the creep curve, introduced as follows.

#### 2.3.2. Constitutive Equation Derived with “Finite” Loading Rate for Analyzing Creep Behavior

The constitutive equation derived with finite loading rate for curvefitting creep curve is derived based on the fact that the stress rate used in the loading process in the creep test is a finite constant. This constitutive equation describes a realistic situation since the stress rate must be a finite constant (no matter how slow or fast it is) in a real creep test.

The solution for describing the strain–time relationship of the loading process, i.e., Equation (8), and the solution for describing the strain–time relationship of the creep process, i.e., Equation (9), are used in the derivation of the constitutive equation considering the stress rate as finite for curvefitting creep curve.

Letting $t=t^{\prime }$ (the time point at the end of the loading process, which is also the time point at the beginning of the creep process) in Equation (8), we can obtain $\varepsilon_{0}$ as a function of $\sigma_{0}$:

(10) $$\begin{array}{ll} \varepsilon_{loading}\left(t^{\prime }\right)=\varepsilon_{0} & =\frac{r}{E_{1}}t^{\prime }+\frac{r}{E_{1}}\left(\tau_{R}-\tau_{C}\right)\left(1-e^{\frac{-t^{\prime }}{\tau_{C}}}\right)\\ & =\frac{\sigma_{0}}{E_{1}}+\frac{r}{E_{1}}\left(\tau_{R}-\tau_{C}\right)\left(1-e^{\frac{-\sigma_{0}}{\tau_{C}\cdot r}}\right) \end{array}$$

In Equation (10), we have used the relationship $\sigma_{0}=rt^{\prime }$, meaning that the stress at the end of the loading process is equal to the stress rate multiplies the time duration of the loading process. Substituting Equation (10) into Equation (9), the strain–time relationship of the creep process can be expressed as:

(11) $$\varepsilon_{creep}\left(t\right)=\left[\frac{r}{E_{1}}\left(\tau_{R}-\tau_{C}\right)\left(1-e^{\frac{-\sigma_{0}}{\tau_{C}\cdot r}}\right)\right]e^{\frac{-t}{\tau_{C}}}+\frac{\sigma_{0}}{E_{1}}$$

If the time-dependent ordinary exponential function in Equation (11), i.e., $e^{-t/\tau_{C}}$, is replaced by a stretched-exponential function $e^{-{\left(t/\tau_{C}\right)}^{k}}$, the curvefitting ability of the constitutive equation can be greatly improved so that the constitutive equation can account for the creep behaviors of most of the materials. By doing so, Equation (11) becomes:

(12) $$\varepsilon_{creep}\left(t\right)=\left[\frac{r}{E_{1}}\left(\tau_{R}-\tau_{C}\right)\left(1-e^{\frac{-\sigma_{0}}{\tau_{C}\cdot r}}\right)\right]e^{-{\left(\frac{t}{\tau_{C}}\right)}^{k}}+\frac{\sigma_{0}}{E_{1}}$$

where k∈ℝ and 0<k≤1. Equation (12) is namely the constitutive equation derived with finite stress rate for curveffing creep curve based on the standard linear solid model. In Equation (12), $E_{1}$, $E_{2}$ and η are three parameters in the standard linear solid model relevant to the viscoelastic properties, and they are unknown variables to be determined in the analysis. The values of r (i.e., the stress rate used in the loading process) and $\sigma_{0}$ (i.e., the constant stress during the creep process) are known since they are preset parameters designated by the tester in the creep test. r and $\sigma_{0}$ can also be obtained from the data of the test.

The practical method of using Equation (12) to analyze creep behavior to obtain the viscoelastic properties of materials is: Using Equation (12) to curvefit the creep curve, the values of $E_{1}$, $\tau_{C}$ and $\tau_{R}$ can be obtained. By solving two simultaneous equations $\tau_{C}=\eta \left(E_{1}+E_{2}\right)/E_{1}E_{2}$ and $\tau_{R}=\eta /E_{2}$, the values of $E_{2}$ and η can be obtained. Therefore, all of the three parameters in the standard linear solid model relevant to the viscoelastic properties, $E_{1}$, $E_{2}$ and η, can be obtained. Please see Supplementary Materials File S2 for a MATLAB computer programming code for curvefitting creep curve based on using Equation (12).

#### 2.3.3. Constitutive Equation Form Derived with “Infinite” Loading Rate for Analyzing Creep Behavior

The constitutive equation derived with infinite loading rate for curvefitting creep curve is derived based on the assumption that the stress rate used in the loading process approaches infinity (i.e., extremely fast):

(13) $$\varepsilon_{creep}\left(t\right)=\frac{\sigma_{0}}{E_{1}}\left(1-\frac{E_{2}}{E_{1}+E_{2}}e^{\frac{-t}{\tau_{C}}}\right)$$

Please refer to Supplementary Materials File S1 for the derivation of Equation (2).

The practical method of using Equation (2) to analyze creep behavior to obtain the viscoelastic properties of materials is: Using Equation (2) to curvefit the creep curve, the values of $E_{1}$, $E_{2}$ and $\tau_{C}$ can be obtained. Since $E_{1}$, $E_{2}$ and $\tau_{C}$ have been obtained, and knowing that $\tau_{C}=\eta \left(E_{1}+E_{2}\right)/E_{1}E_{2}$, the value of η can be obtained. Therefore, all of the three parameters in the standard linear solid model relevant to the viscoelastic properties, $E_{1}$, $E_{2}$ and η, can be obtained. Please see Supplementary Materials File S2 for a MATLAB computer programming code for curvefitting creep curve based on using Equation (2).

### 2.4. Finite Element Computational Simulation

The purpose of the finite element computational simulation is to evaluate the accuracy of the constitutive equations derived with finite and infinite loading rate. The idea is: First, finite element computational simulation will be used to simulate a series of stress relaxation and creep curves tested with different loading rates (from extremely slow to extremely fast). The viscoelastic properties of the material designated in the finite element simulation software suite during the simulation are served as the true viscoelastic properties of the material. Next, each of the simulated stress relaxation and creep curves is curvefitted by the constitutive equations derived with finite and infinite loading rates, respectively. The curvefitting on each simulated curve by each equation form will yield a set of evaluated viscoelastic properties (i.e., analysis results, called the analyzed viscoelastic properties). Finally, the analyzed viscoelastic properties will be compared to the true ones. If the analyzed viscoelastic properties are close to the true ones, the equation form can be validated to be accurate.

Finite element computational simulation is performed using ABAQUS 2019 (Dassault Systems Simulia Corporation, Johnson, RI, USA). In the finite element computational simulation, an axisymmetric model with radius of 5 mm and thickness of 10 mm is used. A total of 5000 quadrilateral elements (0.1 mm × 0.1 mm) and 5151 nodes are used to mesh the model. The boundary conditions are that the top and sides of the model are not fixed (i.e., displacements and rotations are allowed along all directions) while the bottom is fixed along the depth direction (i.e., displacements are not allowed along the depth direction while rotations are not allowed along the lateral direction).

The material that makes up the model is linearly viscoelastic. The material is also assumed to be incompressible, isotropic, and homogeneous. The mechanical properties of the material are defined by four parameters, including the modulus of elasticity (E), Poisson’s ratio (set as 0.495, the maximum Poisson’s ratio that can be set in ABAQUS), g and $\tau_{R}$, which are two parameters in the one-branch dimensionless relaxation modulus:

(14) $$g_{R}\left(t\right)=1-g\left(1-e^{\frac{-t}{\tau_{R}}}\right)$$

where g is a material constant (0<g<1), and $\tau_{R}$ is the relaxation time constant. It has been reported that $E_{1}$ of the standard linear solid model is equal to E, while g is equal to $E_{2}/\left(E_{1}+E_{2}\right)$ and $\tau_{R}$ is equal to $\eta /E_{2}$ [8]. From the equation $g=E_{2}/\left(E_{1}+E_{2}\right)$, it can be deduced that $E_{2}=Eg/\left(1-g\right)$. Then, from the equation $\tau_{R}=\eta /E_{2}$, it can be deduced that $\eta =\tau_{R}E_{2}=\tau_{R}Eg/\left(1-g\right)$. Therefore, there exists a relationship between the three mechanical properties defined in ABAQUS (i.e., E, g and $\tau_{R}$) and the three parameters in the standard linear solid model (i.e., $E_{1}$, $E_{2}$ and η). By using this relationship, E, g and $\tau_{R}$ can be transformed into $E_{1}$, $E_{2}$ and η, and vice versa.

Nine material models with different mechanical properties are analyzed, and the setting of their mechanical properties are shown in Table 1. The material models are assumed to be hydrogels that mimic soft tissues; therefore, the values of the modulus of elasticity [85] and the relaxation time constant [86] defined for the material models are within the range of reported values for soft tissues.

**Figure 4:**
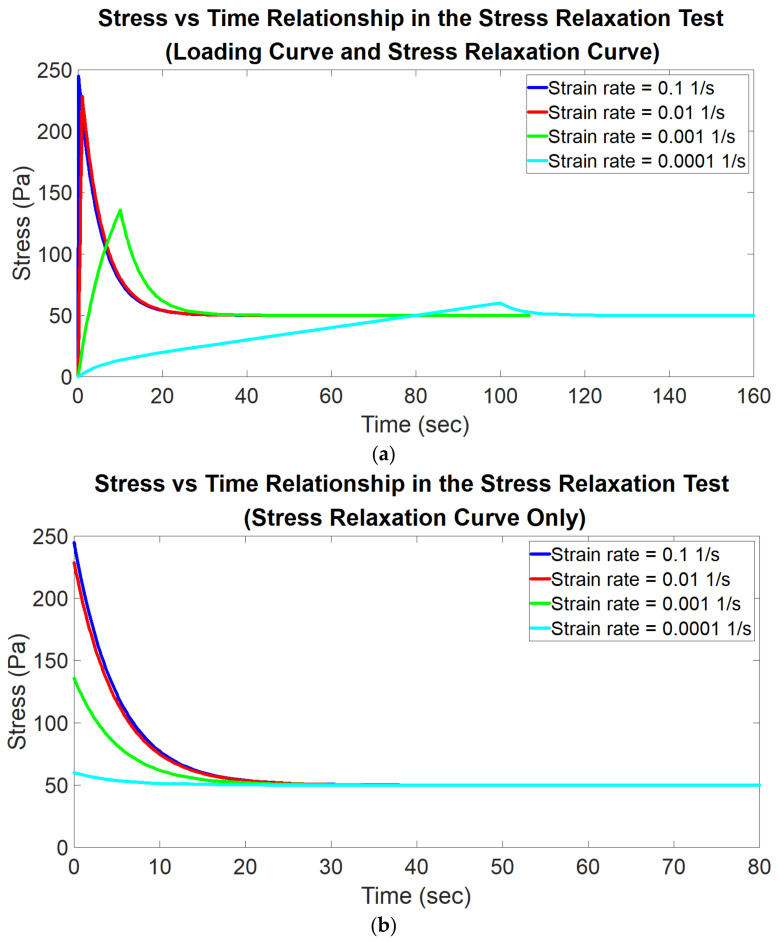
Illustration of an example of a series of simulated stress relaxation curves with associated loading curves measured with different strain rates. (**a**) Loading curve and stress relaxation curve. (**b**) Stress relaxation curve only.

In the simulation of stress relaxation behavior, a uniform deformation is applied to the top of the model. For each material model, the strain rate used in the loading process is respectively set as 0.0001, 0.001, 0.01, 0.1, 1, 10, and 100 1/s (the corresponding time interval in the loading process is 100, 10, 1, 0.1, 0.01, 0.001, and 0.0001 s, respectively); in other words, each material model is tested with these seven strain rates respectively. Since there are nine material models, there is a total of 63 simulation trails in the simulation of stress relaxation behavior. Once the maximum deformation of 0.1 mm is reached, the deformation is then maintained as constant at 0.1 mm for a period of time. During this period, each element in the model exhibits stress relaxation behavior; that is, the stress of each element decreases with time until a constant stress level is reached. Figure 4 shows an example of a series of simulated stress relaxation curves with associated loading curves tested using different strain rates. The stress and strain (along the depth direction) versus time data of each element are recorded and then imported into MATLAB for analysis. The two equation forms, i.e., Equations (1) and (7), are used respectively to curvefit the stress relaxation curve of each element for obtaining the corresponding $E_{1}$, $E_{2}$ and η. Consequently, for each equation form, each element is associated with a specific set of $E_{1}$, $E_{2}$ and η. For each equation form, the average of the values of all of the elements in each viscoelastic property ($E_{1}$, $E_{2}$ and η) is defined as the analyzed viscoelastic property, which is compared to the true viscoelastic property using the following equation:

(15) $$error=\frac{\left|analyzed\;viscoelastic\;property-true\;viscoelastic\;property\right|}{theoretical\;viscoelastic\;property}$$

If the error is less than 5%, the evaluation is considered to be accurate. The error margin of 5% is chosen in our analyses because it is an error margin commonly used in various fields of scientific research according to literatures [87,88,89,90,91,92,93,94]. For each viscoelastic property ($E_{1}$, $E_{2}$ and η), the relationship between the strain rate and the error analyzed by each equation form is investigated.

**Figure 5:**
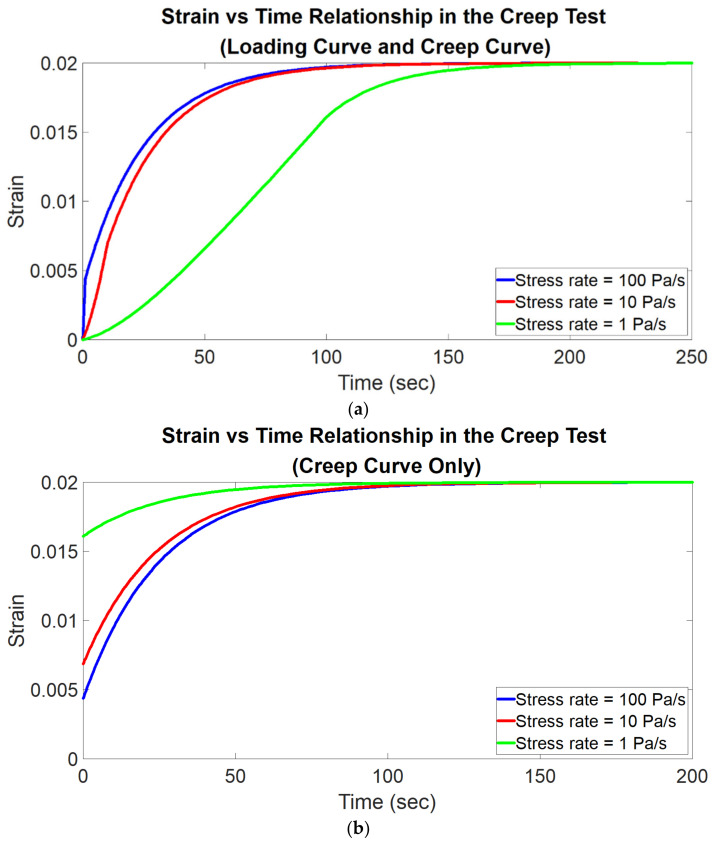
Illustration of an example of a series of simulated creep curves with associated loading curves measured with different stress rates. (**a**) Loading curve and creep curve. (**b**) Creep curve only.

In the simulation of creep behavior, a uniform pressure is applied to the top of the model. For each material model, the stress rate used in the loading process is respectively set as 1, 10, 100, 1000, 10,000, 100,000, 1,000,000 Pa/s (the corresponding time interval in the loading process is 100, 10, 1, 0.1, 0.01, 0.001 and 0.0001 s respectively); in other words, each material model is tested with these seven stress rates respectively. Since there are nine material models, there is a total of 63 simulation trails in the simulation of creep behavior. Once the maximum pressure of 100 Pa is reached, the pressure is then maintained as constant at 100 Pa for a period of time. During this period, each element in the model exhibits creep behavior; that is, the strain of each element increases with time until a constant strain level is reached. Figure 5 shows an example of a series of simulated creep curves with associated loading curves tested using different stress rates. The stress and strain (along the depth direction) versus time data of each element are recorded and then imported into MATLAB for analysis. The two equation forms, i.e., Equations (2) and (12), are used respectively to curvefit the creep curve of each element for obtaining the corresponding $E_{1}$, $E_{2}$ and η. The following data analysis method is the same as that described above in the simulation of stress relaxation behavior.

## 3. Results

### 3.1. Results: Stress Relaxation

**Figure 6:**
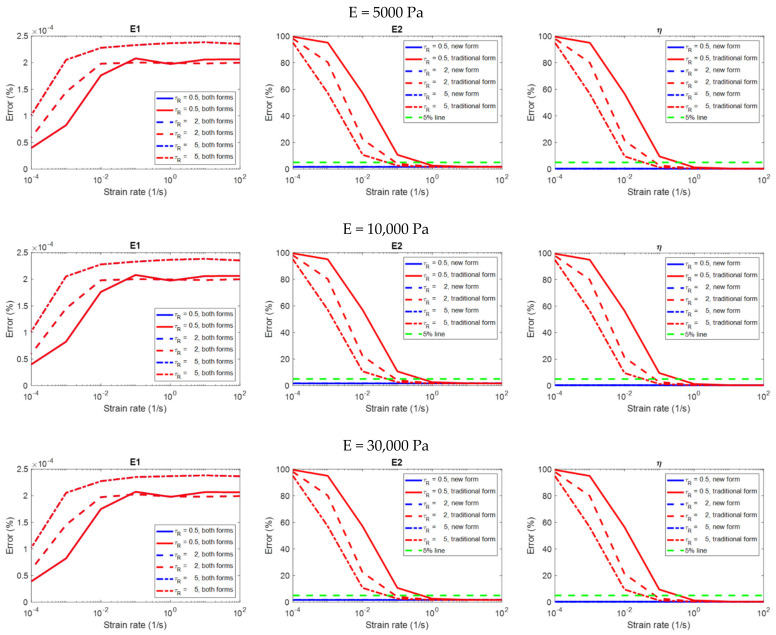
The relationship between the strain rate and the error in the evaluation of the viscoelastic properties on different material models in the stress relaxation simulation, analyzed using the two constitutive equation forms (i.e., the newly−introduced form derived with finite loading rate, and the traditional form derived with infinite loading rate).

**Figure 7:**
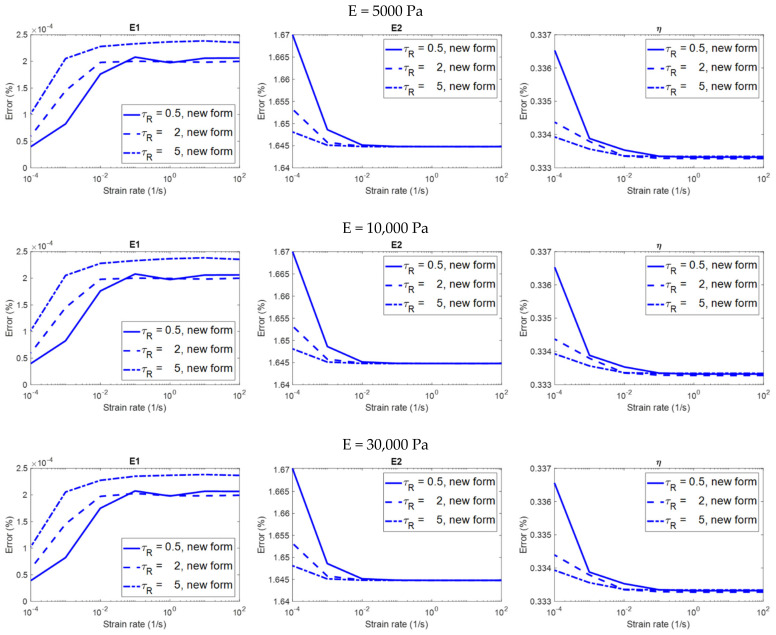
The relationship between the strain rate and the error in the evaluation of the viscoelastic properties on different material models in the stress relaxation simulation, analyzed using the newly−introduced constitutive equation derived with finite loading rate.

Figure 6 shows the relationship between the strain rate and the error in the evaluation of the viscoelastic properties on different material models analyzed using the two equation forms. Figure 7 only shows the results analyzed using the constitutive equation derived with a finite loading rate in order to highlight the details. Please remember that the evaluation is considered to be accurate if the error is less than 5%. Some observations on the analysis results that are more relevant to practical applications are summarized below:

(1) The constitutive equation derived with a finite loading rate can always produce accurate results in the evaluation of all of the viscoelastic properties $E_{1}$, $E_{2}$ and η, regardless of the strain rate (Figure 6 and Figure 7). In the evaluation of $E_{1}$, $E_{2}$ and η, the results are dependent on both the strain rate and the relaxation time constant of the material; some significant trends can be observed (Figure 7). However, these dependencies might not be important for practical applications since the errors are already very low regardless of the strain rate, and the error values at different strain rates do not differ significantly.
(2) For the constitutive equation derived with infinite loading rate in the evaluation of $E_{2}$ and η, the higher the strain rate, the more accurate the result is (the second and third columns in Figure 6). This equation form can produce accurate evaluations if and only if when the strain rate is higher than a threshold (which is the strain rate value at the intersection of the green dotted line and the red curves in Figure 6). It can be observed that the threshold is dependent on the relaxation time constant of the material; the lower the relaxation time constant, the higher the threshold.
(3) In the evaluation of $E_{1}$, the two equation forms can produce accurate and identical evaluations regardless of the strain rate (the first column in Figure 6). The results are dependent on both the strain rate and the relaxation time constant; some significant trends can be observed. However, these dependencies might not be important for practical applications since the errors are already very low regardless of the strain rate, and the error values at different strain rates do not differ significantly.
(4) No matter what the modulus of elasticity (E) of the material is (5000, 10,000, or 30,000 Pa), the analysis results are identical (three rows in Figure 6). That is to say, the analysis results are independent of the modulus of elasticity.

### 3.2. Results: Creep

**Figure 8:**
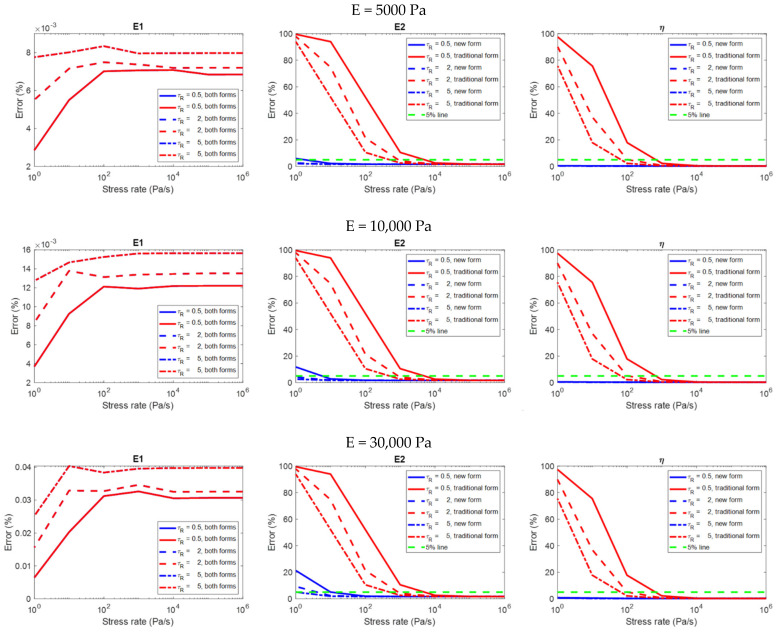
The relationship between the stress rate and the error in the evaluation of the viscoelastic properties on different material models in the creep simulation, analyzed using the two constitutive equation forms (i.e., the newly−introduced form derived with finite loading rate, and the traditional form derived with infinite loading rate).

**Figure 9:**
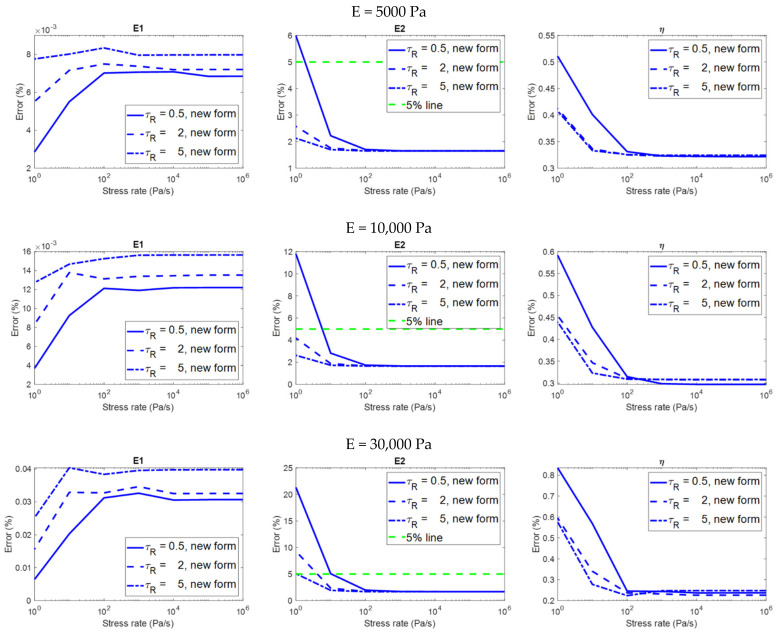
The relationship between the stress rate and the error in the evaluation of the viscoelastic properties on different material models in the creep simulation, analyzed using the newly−introduced constitutive equation derived with finite loading rate.

Figure 8 shows the relationship between the stress rate and the error in the evaluation of the viscoelastic properties on different material models analyzed using the two equation forms. Figure 9 only shows the results analyzed using the constitutive equation derived with a finite loading rate in order to highlight the details. Please remember that the evaluation is considered to be accurate if the error is less than 5%. Some observations on the analysis results that are more relevant to practical applications are summarized below:

(1) The constitutive equation derived with a finite loading rate can always produce accurate results in the evaluation of $E_{1}$ and η, regardless of the stress rate (Figure 8 and Figure 9). However, in the evaluation of $E_{2}$, this equation form can produce accurate results if and only if when the stress rate is higher than a threshold (which is the stress rate value at the intersection of the green dotted line and the red curves in Figure 9). It can be observed that the threshold is dependent on the relaxation time constant of the material; the lower the relaxation time constant, the higher the threshold.
(2) For the constitutive equation derived with infinite loading rate in the evaluation of $E_{2}$ and η, the higher the stress rate, the more accurate the result is (the second and third columns in Figure 8). This equation form can produce accurate evaluations if and only if when the stress rate is higher than a threshold (which is the stress rate value at the intersection of the green dotted line and the red curves in Figure 8). It can be observed that the threshold is dependent on the relaxation time constant of the material; the lower the relaxation time constant, the higher the threshold.
(3) In the evaluation of $E_{1}$, the two equation forms can produce accurate and identical evaluations regardless of the stress rate (the first column in Figure 8). The results are dependent on both the stress rate and the relaxation time constant; some significant trends can be observed. However, these dependencies might not be important for practical applications since the errors are already very low regardless of the stress rate, and the error values at different stress rates do not differ significantly.
(4) The analysis results are dependent on the modulus of elasticity (E) of the material. Generally speaking, the higher the modulus of elasticity, the larger the error (three rows in Figure 8).

## 4. Discussion

This paper introduces the constitutive equations for analyzing stress relaxation and creep behaviors based on the standard linear solid model derived with finite loading rate, i.e., Equations (7) and (12). The finite element computational simulation results demonstrate that, by using these constitutive equations to curvefit stress relaxation and creep curves, they can produce accurate results in the evaluation of all of the viscoelastic properties $E_{1}$, $E_{2}$ and η regardless of the loading rate used in the loading process of the stress relaxation and creep tests in most cases (the exceptions will be discussed in the next paragraph).

The viscoelastic properties are intrinsic properties of a material, therefore should be constant and independent of the loading rate used for testing. Hence, theoretically speaking, if a constitutive equation is derived with a finite loading rate, the analyzed viscoelastic properties by that constitutive equation should be constant and independent of the loading rate. Indeed, it is true that the constitutive equation derived with a finite loading rate for stress relaxation can always produce accurate results in the evaluation of all of the viscoelastic properties $E_{1}$, $E_{2}$ and η regardless of the strain rate. However, surprisingly, the constitutive equation derived with a finite loading rate for creep cannot be universally applied to all creep behaviors tested with any stress rate. Specifically, although it can always produce accurate results in the evaluation of $E_{1}$ and η regardless of the stress rate, it cannot produce accurate results in the evaluation of $E_{2}$ if the stress rate is lower than a threshold. It means that if the constitutive equation derived with a finite loading rate for creep is intended to be applied to analyze a creep curve, the stress rate used in the loading process of the creep test for obtaining that creep curve must be higher than the threshold to yield an accurate evaluation for all of the viscoelastic properties. Based on the results of the present study on a limited range of data, a stress rate higher than 100 Pa/s can ensure an accurate evaluation. Unfortunately, this threshold value is typically unknown in a real experiment when the viscoelastic properties of a material are unknown and intended to be determined since this threshold depends on the viscoelastic properties, including the relaxation time constant and the modulus of elasticity, as the findings of the present study have shown. The reason why the constitutive equation derived with a finite loading rate for creep cannot be universally applied to all creep behaviors tested with any stress rate can be understood by examining Equation (12). If r (i.e., the stress rate) in Equation (12) approaches zero, the first term right to the equality sign (i.e., the term involving the time-dependent exponential function that characterizes the time-dependent viscoelastic behavior) approaches zero, and only the second term (i.e., the constant term that characterizes the time-independent elastic solid behavior) remains. It means that if the stress rate is very low, the viscoelastic material behaves more similar to an elastic solid and displays only a few or no viscoelastic behaviors. The evidence can be observed in Figure 5 that when the stress rate is very low, the pattern of the creep curve is relatively insignificant. Therefore, if the constitutive equation derived with a finite loading rate for creep is applied to analyze a creep curve tested with an extremely low stress rate, an accurate evaluation might not be obtained. The same problem might occur in the analysis of stress relaxation but at a strain rate lower than the minimum strain rate investigated in the present study. Therefore, such a problem is not observed in the analysis of stress relaxation in the present study. In order to avoid this problem in practice, we suggest that a relatively higher stress rate (or strain rate) should be used in the loading process of a creep test (or stress relaxation test), and the relevant constitutive equation derived with finite loading rate is then applied for the analysis.

The present study shows that the traditional constitutive equations derived with infinite loading rate for both stress relaxation and creep can produce accurate results in the evaluation of $E_{2}$ and η if and only if when the loading rate is higher than a threshold. The higher the loading rate, the more accurate the evaluation. On the other hand, if the loading rate is lower than the threshold, the evaluation can be very inaccurate (the error can be up to near 100% if the loading rate is very low). Such findings are expectable. Since these constitutive equations are derived with an infinite loading rate, they are just approximations and can only be applied to analyze data acquired with extremely fast loading rates. Since our proposed constitutive equations derived with finite loading rate can be universally applied to all stress relaxation and creep behaviors regardless of the loading rate in most cases, it is recommended that our proposed equations should replace the traditional equations to analyze stress relaxation and creep behaviors for quantitatively evaluating the viscoelastic properties of materials. Compared to using the constitutive equations derived with finite loading rate, there could be no benefits from using the traditional equations derived with infinite loading rate.

In the evaluation of $E_{1}$, it is interesting to note that the two constitutive equation forms can produce accurate and identical evaluations regardless of the loading rate. This finding can be applied to both stress relaxation and creep. The reason why the evaluation of $E_{1}$ is independent of the equation form used for the analysis is that the physical meaning of $E_{1}$ is the modulus of elasticity of the material [8], which is a solid elastic property independent of the loading rate. Since the fundamental difference between the two equation forms is their dependencies on the loading rate, the two equation forms can produce accurate and identical results in the evaluation of the loading-rate-independent property $E_{1}$. On the other hand, since both $E_{2}$ and η are properties relevant to viscous fluids that are dependent on the loading rate, their evaluations must be dependent on the equation form used for the analysis.

The physical significances of our proposed constitutive equations derived with finite loading rate and the traditional constitutive equations derived with infinite loading rate are summarized in Table 2 (for stress relaxation) and Table 3 (for creep). In each equation, the term involving the time-dependent exponential function characterizes the time-dependent viscoelastic behavior, while the constant term characterizes the time-independent elastic solid behavior. It can be observed that the constant term characterizing the time-independent elastic solid behavior is the same for both equation forms. In addition, the initial mechanical response at t=0 for the traditional equation is a constant, while that for our proposed equation is positively correlated with r. It means that, in a real experiment, the larger the loading rate, the larger the initial mechanical response at t=0, as predicted by our proposed equation. These observations can be applied to both stress relaxation and creep.

Using an accurate solution form of the standard linear solid model for the analysis is important for successful applications. For example, the standard linear model has been applied in the development of an ultrasound imaging technique called ultrasound viscoelastic creep imaging, which aims to quantitatively evaluate the internal spatial distributions of the viscoelastic properties of materials [9,10,95]. In ultrasound viscoelastic creep imaging, a constant uniaxial stress field is produced within the material using either acoustic radiation force or mechanical compression to induce the creep behavior of the material, and then ultrasound imaging is used to measure the creep curve of each pixel of the image. By applying an appropriate solution form of the standard linear solid model to curvefit the creep curve of each pixel, the viscoelastic properties of each pixel can be quantitatively evaluated. Then, by collecting the viscoelastic properties of every pixel of the image, the two-dimensional spatial distribution maps of the viscoelastic properties of a cross section of the material can be obtained. Based on the present study, there are many forms of solution for the standard linear solid model, and choosing the most accurate one for the analysis in ultrasound viscoelastic creep imaging is important for obtaining accurate maps of the viscoelastic properties and ensuring the authenticity of the further analyses. The resulting images of the viscoelastic properties obtained using ultrasound viscoelastic creep imaging can be useful for researchers and practitioners to apply in many fields, such as rehabilitation [96]. For instance, with the evaluated viscoelastic properties of tissues, therapists can treat them as a reliable index to objectively evaluate the condition of tissues and diagnose the severity of injuries of tissues. In addition, by monitoring the viscoelastic properties of tissues during the recovery process, therapists can use them as the basis for assessing the effect of rehabilitation since one of the aims of many rehabilitation strategies focuses on inducing positive changes in the viscoelastic properties of tissues. Based on such information, therapists can select the best rehabilitation strategy by monitoring the responses of viscoelastic properties to interventions for a specific patient with functional impairments. Furthermore, quantitative evaluation of the viscoelastic properties of tissues can help to identify new rehabilitation strategies.

The main limitation of the present study is that only a limited number of viscoelastic properties and loading rates can be investigated in the finite element computational simulation. In the future, it is important to acquire a larger data set by investigating a wider range of viscoelastic properties and loading rates in order to investigate the generality and validity of our proposed constitutive equations derived with a finite loading rate.

## 5. Conclusions

This paper introduces the constitutive equations for analyzing stress relaxation and creep behaviors based on the standard linear solid model derived with a finite loading rate, i.e., Equations (7) and (12). The finite element computational simulation results show that, by using those constitutive equations to analyze stress relaxation and creep curves, they can produce accurate results in the evaluation of all of the viscoelastic properties $E_{1}$, $E_{2}$, and η regardless of the loading rate used in the loading process of the stress relaxation and creep tests in most cases. Since the constitutive equations derived with a finite loading rate can be universally applied to all stress relaxation behaviors tested with any strain rate and can be applied to all creep behaviors tested with any stress rate except for those tested with extremely slow stress rates, it is recommended that the constitutive equations derived with a finite loading rate should replace the traditional equations derived from an infinite loading rate to analyze stress relaxation and creep behaviors for quantitatively evaluating the viscoelastic properties of materials.

## Figures and Tables

**Table 1:** The setting of the mechanical properties of the nine material models used in the simulation.

	Mechanical Properties Set in ABAQUS		Corresponding Parameters in the Standard Linear Solid Model			
Number of Material Models	E (kPa)	$\tau_{R}$ (s)	g	$E_{1}$ (kPa)	$E_{2}$ (kPa)	η (Pa · s)
1	5	0.5	0.8	5	20	10
2	5	2	0.8	5	20	40
3	5	5	0.8	5	20	100
4	10	0.5	0.8	10	40	20
5	10	2	0.8	10	40	80
6	10	5	0.8	10	40	200
7	30	0.5	0.8	30	120	60
8	30	2	0.8	30	120	240
9	30	5	0.8	30	120	600

**Table 2:** Physical significances of our proposed constitutive equations derived with finite loading rate, i.e., Equation (7), and the traditional equations derived with infinite loading rate, i.e., Equation (1), for stress relaxation.

Physical Significances	Equation (1)	Equation (7)
The term involving the time-dependent exponential function that characterizes the time-dependent viscoelastic behavior	$E_{2}\varepsilon_{0}e^{\frac{-t}{\tau_{R}}}$	$\left[E_{1}r\left(\tau_{C}-\tau_{R}\right)\left(1-e^{\frac{-\varepsilon_{0}}{\tau_{R}\cdot r}}\right)\right]e^{-{\left(\frac{t}{\tau_{R}}\right)}^{k}}$
The constant term that characterizes the time-independent elastic solid behavior (which is also the equilibrium stress when t→∞)	$E_{1}\varepsilon_{0}$	$E_{1}\varepsilon_{0}$
The initial stress at the beginning of the stress relaxation process at t=0)	$\left(E_{1}+E_{2}\right)\varepsilon_{0}$	$E_{1}r\left(\tau_{C}-\tau_{R}\right)\left(1-e^{\frac{-\varepsilon_{0}}{\tau_{R}\cdot r}}\right)+E_{1}\varepsilon_{0}$

**Table 3:** Physical significances of our proposed constitutive equations derived with finite loading rate, i.e., Equation (12), and the traditional equations derived with infinite loading rate, i.e., Equation (2), for creep.

Physical Significances	Equation (2)	Equation (12)
The term involving the time-dependent exponential function that characterizes the time-dependent viscoelastic behavior	$-\frac{E_{2}}{E_{1}+E_{2}}\frac{\sigma_{0}}{E_{1}}e^{\frac{-t}{\tau_{C}}}$	$\left[\frac{r}{E_{1}}\left(\tau_{R}-\tau_{C}\right)\left(1-e^{\frac{-\sigma_{0}}{\tau_{C}\cdot r}}\right)\right]e^{-{\left(\frac{t}{\tau_{C}}\right)}^{k}}$
The constant term that characterizes the time-independent elastic solid behavior (which is also the equilibrium strain when t→∞)	$\frac{\sigma_{0}}{E_{1}}$	$\frac{\sigma_{0}}{E_{1}}$
The initial strain at the beginning of the creep process at t=0)	$\frac{\sigma_{0}}{E_{1}+E_{2}}$	$\left[\frac{r}{E_{1}}\left(\tau_{R}-\tau_{C}\right)\left(1-e^{\frac{-\sigma_{0}}{\tau_{C}\cdot r}}\right)\right]+\frac{\sigma_{0}}{E_{1}}$

## Data Availability

The datasets relevant to the present study are available on request to the corresponding author.

## Supplementary Materials

The following supporting information can be downloaded at: https://www.mdpi.com/article/10.3390/polym14102124/s1, File S1: Supplementary Material 1; File S2: Supplementary Material 2.
