# Supplementary material for: Constitutive Equations for Analyzing Stress Relaxation and Creep of Viscoelastic Materials Based on Standard Linear Solid Model Derived with Finite Loading Rate
Source: Polymers (Basel). 2022 May 23;14(10):2124. doi: 10.3390/polym14102124 (PMC9143375; doi:10.3390/polym14102124)
Supplement: Supplementary file 1 [file polymers-14-02124-s001.zip › Supplementary Material 1.pdf]

Supplementary Material 1

Title of the paper: Constitutive equations for analyzing stress relaxation and creep of viscoelastic materials based on standard linear solid model derived with finite loading rate

Authors: Che-Yu Lin [1,*], Yi-Cheng Chen [1], Chen-Hsin Lin [2] and Ke-Vin Chang [3,4,5,†]
1 Institute of Applied Mechanics, College of Engineering, National Taiwan University, No. 1, Sec. 4, Roo-sevelt Road, Taipei 10617, Taiwan
2 Department of Mechanical Engineering, College of Engineering, National Taiwan University, No. 1, Sec. 4, Roosevelt Road, Taipei 10617, Taiwan
3 Department of Physical Medicine and Rehabilitation and Community and Geriatric Research Center, National Taiwan University Hospital, Bei-Hu Branch, Taipei, Taiwan
4 Department of Physical Medicine and Rehabilitation, National Taiwan University College of Medicine, Taipei, Taiwan
5 Center for Regional Anesthesia and Pain Medicine, Wang-Fang Hospital, Taipei Medical University, Taipei, Taiwan
* Correspondence: cheyu@ntu.edu.tw
† Co-corresponding author.

Purpose of the Supplementary Material 1: This supplementary material provides the derivations of some equations appeared in the paper.

Table of Content of the Supplementary Material 1:

1. Derivation of the constitutive equation of the Maxwell form of the standard linear solid model Page.2
2. Derivation of the constitutive equation of the Maxwell model Page.4
3. Derivation of Equations (1) and (2) in the paper Page.6
4. Derivation of Equations (3) and (4) in the paper Page.9
5. Derivation of Equations (8) and (9) in the paper Page.12

1. Derivation of the constitutive equation of the Maxwell form of the standard linear solid model

In this section, we are going to introduce the derivation of the constitutive equation of the Maxwell form of the standard linear solid model (abbreviated as the standard linear solid model in the following text).

The standard linear solid model is a three-element model consisting of a linear spring and a Maxwell model in parallel (Fig. S1). Please note that, the Maxwell model is the upper arm of the standard linear solid model, and it is a two-element model consisting of a linear spring and a linear dashpot in series.

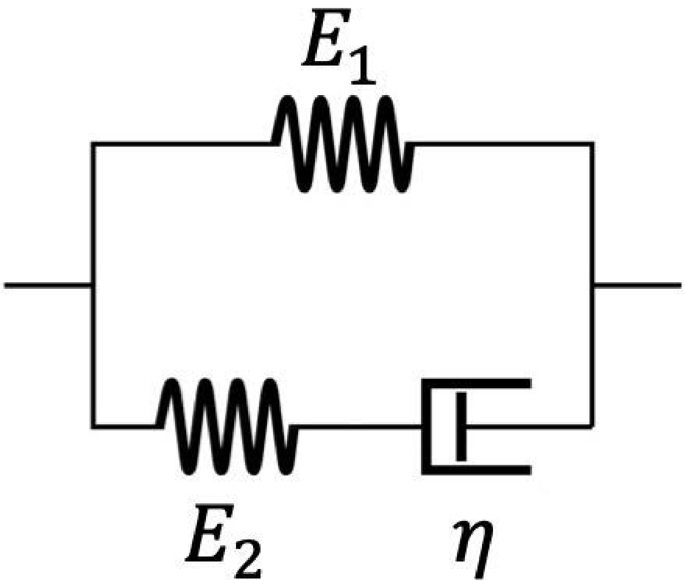

Figure S1 The Maxwell form of the standard linear solid model. E_1, E_2 and η are three parameters in the standard linear solid model relevant to the viscoelastic properties.

If an external load is applied to the standard linear solid model, the model will experience both stress and strain. The strain of the entire model follows the relationship:

$$\varepsilon = \varepsilon_{s1} = \varepsilon_{s2} + \varepsilon_d \tag{S.1}$$

where ε is the strain of the entire model, ε_{s1} is the strain of the spring 1, ε_{s2} is the strain of the spring 2, and ε_d is the strain of the dashpot.

For the spring 1, the Hooke's law can be used to describe the relationship between the stress and strain of this spring:

$$\sigma_{s1} = E_1 \varepsilon_{s1} = E_1 \varepsilon \tag{S.2}$$

where σ_{s1} is the stress of the spring 1, and E_1 is the modulus of elasticity of the spring 1. The second equality in Equation (S.2) holds since the strain of the spring 1 (ε_{s1}) is equal to the strain of the entire model (ε), from Equation (S.1).

Similarly, for the spring 2, the Hooke's law can be used to describe the relationship between the stress and strain of this spring:

$$\sigma_{s2} = E_2 \varepsilon_{s2} \tag{S.3}$$

where σ_{s2} is the stress of the spring 2, and E_2 is the modulus of elasticity of the spring 2.

For the dashpot, the constitutive equation of the linear dashpot can be used to describe the relationship between the stress and strain of this dashpot:

$$\sigma_d = \eta \dot{\varepsilon}_d \tag{S.4}$$

where σ_d is the stress of the dashpot, and η is the viscosity of the dashpot.

The stress of the entire model is the sum of the stresses in the lower and upper arms of the entire model:

$$\sigma = \sigma_{s1} + \sigma_{s2} \tag{S.5}$$

where σ is the stress of the entire model, σ_{s1} is the stress of the spring 1 which is also the stress of the lower arm of the entire model, and σ_{s2} is the stress of the spring 2 which is also the stress of the upper arm of the entire model.

Since the spring 2 and dashpot are connected in series, the stress of the spring 2 (σ_{s2}) is equal to the stress of the dashpot (σ_d), therefore Equation (S.5) can be written as:

$$\sigma = \sigma_{s1} + \sigma_d \tag{S.6}$$

Substituting Equations (S.2) and (S.3) into Equation (S.5):

$$\sigma = E_1 \varepsilon + E_2 \varepsilon_{s2} \tag{S.7}$$

Replacing ε_{s2} in Equation (S.7) by $\varepsilon - \varepsilon_d$ from Equation (S.1):

$$\sigma = E_1 \varepsilon + E_2 (\varepsilon - \varepsilon_d) = (E_1 + E_2)\varepsilon - E_2 \varepsilon_d \tag{S.8}$$

Differentiating Equation (S.8) with time:

$$\dot{\sigma} = (E_1 + E_2)\dot{\varepsilon} - E_2 \dot{\varepsilon}_d \tag{S.9}$$

Replacing $\dot{\varepsilon}_d$ in Equation (S.9) by σ_d/η from Equation (S.4), then replacing this σ_d by $\sigma - \sigma_{s1}$ from Equation (S.6), and then replacing this σ_{s1} by $E_1 \varepsilon$ from Equation (S.2):

$$\dot{\sigma} = (E_1 + E_2)\dot{\varepsilon} - E_2 \frac{\sigma - E_1 \varepsilon}{\eta} \tag{S.10}$$

Equation (S.10) can be rearranged as:

$$\sigma + \tau_R \dot{\sigma} = E_1 (\varepsilon + \tau_C \dot{\varepsilon}) \tag{S.11}$$

where $\tau_R = \eta/E_2$ is the relaxation time constant, and $\tau_C = \eta(E_1 + E_2)/E_1 E_2$ is the creep time constant.

Equation (S.11) is namely the constitutive equation of the standard linear solid model.

2. Derivation of the constitutive equation of the Maxwell model

The constitutive equation of the Maxwell model (Fig. S2) will be used in the derivation of Equations (1) and (2) in the paper, which will be introduced later in this supplementary material. Therefore, in this section, we are going to introduce the derivation of the constitutive equation of the Maxwell model.

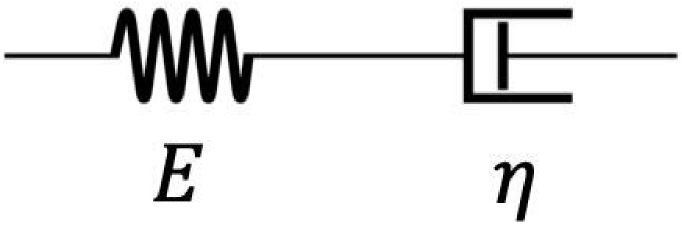

Figure S2 The Maxwell model. E and η are two parameters in the Maxwell model relevant to the viscoelastic properties.

The Maxwell model is a two-element model consisting of a linear spring and a linear dashpot in series. Since both elements are connected in series, the stresses and strains of the spring, dashpot and entire model follow the relationship:

$$\sigma = \sigma_s = \sigma_d \tag{S.12}$$

$$\varepsilon = \varepsilon_s + \varepsilon_d \tag{S.13}$$

where σ and ε are the stress and strain of the entire model respectively, σ_s and ε_s are the stress and strain of the spring respectively, and σ_d and ε_d are the stress and strain of the dashpot respectively.

For the spring, the Hooke's law can be used to describe the relationship between the stress and strain of this spring:

$$\sigma_s = E\varepsilon_s \Longrightarrow \varepsilon_s = \frac{\sigma}{E} \tag{S.14}$$

where E is the modulus of elasticity of the spring.

For the dashpot, the constitutive equation of the linear dashpot can be used to describe the relationship between the stress and strain of this dashpot:

$$\sigma_d = \eta\dot{\varepsilon}_d \Longrightarrow \dot{\varepsilon}_d = \frac{\sigma}{\eta} \Longrightarrow \varepsilon_d = \int \frac{\sigma}{\eta} dt \tag{S.15}$$

where η is the viscosity of the dashpot.

Equation (S.12) has been used in Equations (S.14) and (S.15) for replacing σ_s and σ_d by σ.

Substituting Equations (S.14) and (S.15) into Equation (S.13):

$$\varepsilon = \varepsilon_s + \varepsilon_d \Longrightarrow \varepsilon = \frac{\sigma}{E} + \int \frac{\sigma}{\eta} dt \tag{S.16}$$

Differentiating Equation (S.16) with time:

$$\dot{\varepsilon} = \frac{\dot{\sigma}}{E} + \frac{\sigma}{\eta} \tag{S.17}$$

Equation (S.17) can be rearranged as:

$$\sigma + \frac{\eta}{E}\dot{\sigma} = \eta\dot{\varepsilon} \tag{S.18}$$

Equation (S.18) is namely the constitutive equation of the Maxwell model.

3. Derivation of Equations (1) and (2) in the paper

In this section, we are going to introduce the derivation of Equations (1) and (2) in the paper. The method of Laplace transform is used in the following derivations. Please note that, since the initial stress and strain of a mechanical element (spring or dashpot) in the standard linear solid model are assumed to be zero, the Laplace transform of the first time derivative of a stress or strain function is the Laplace variable s times the Laplace transform of that stress or strain function, such as $\mathcal{L}\{\dot{\sigma}(t)\} = s\mathcal{L}\{\sigma(t)\} = s\bar{\sigma}$ and $\mathcal{L}\{\dot{\varepsilon}(t)\} = s\mathcal{L}\{\varepsilon(t)\} = s\bar{\varepsilon}$.

In the standard linear solid model shown in Figure S1, the spring 1 in the lower arm and the Maxwell model in the upper arm experience the same strain since they are in parallel, and the strain they experience is equal to the strain of the entire model. For the spring 1, the Hooke's law can be used to describe the relationship between the stress and strain of this spring:

$$\sigma_1 = E_1\varepsilon_1 = E_1\varepsilon \tag{S.19}$$

where σ_1 is the stress of the spring 1, E_1 is the modulus of elasticity of the spring 1, and ε_1 is the strain of the spring 1 which is equal to the strain of the entire model ε.

Taking the Laplace transform of Equation (S.19):

$$\overline{\sigma}_1 = E_1\overline{\varepsilon} \tag{S.20}$$

For the Maxwell model, the constitutive equation of the Maxwell model can be used to describe the relationship between the stress and strain of this Maxwell model:

$$\sigma_2 + \frac{\eta}{E_2}\dot{\sigma}_2 = \eta\dot{\varepsilon}_2 = \eta\dot{\varepsilon} \tag{S.21}$$

where σ_2 is the stress of the Maxwell model, η is the viscosity of the dashpot in the Maxwell model, E_2 is the modulus of elasticity of the spring in the Maxwell model, and ε_2 is the strain of the Maxwell model which is equal to the strain of the entire model ε.

Taking the Laplace transform of Equation (S.21):

$$\overline{\sigma}_2 + \frac{\eta}{E_2}s\overline{\sigma}_2 = \eta s\overline{\varepsilon} \implies \overline{\sigma}_2 + \tau_R s\overline{\sigma}_2 = \tau_R E_2 s\overline{\varepsilon} \implies \overline{\sigma}_2 = \frac{E_2 s}{s + \frac{1}{\tau_R}}\overline{\varepsilon} \tag{S.22}$$

where $\tau_R = \eta/E_2$ is the relaxation time constant.

The stress of the entire model is the sum of the stress of the spring 1 and the stress of the Maxwell model:

$$\sigma = \sigma_1 + \sigma_2 \tag{S.23}$$

where σ is the stress of the entire model.

Taking the Laplace transform of Equation (S.23):

$$\overline{\sigma} = \overline{\sigma}_1 + \overline{\sigma}_2 \tag{S.24}$$

Substituting Equations (S.20) and (S.22) into Equation (S.24):

$$\overline{\sigma} = \overline{\sigma}_1 + \overline{\sigma}_2 = E_1\overline{\varepsilon} + \frac{E_2 s}{s + \frac{1}{\tau_R}}\overline{\varepsilon} = \left(E_1 + \frac{E_2 s}{s + \frac{1}{\tau_R}}\right)\overline{\varepsilon} \tag{S.25}$$

In the stress relaxation test using a step function as the loading, $\varepsilon(t)$ is equal to a constant strain value ε_0 times the unit step function:

$$\varepsilon(t) = \varepsilon_0 u(t), \text{ where } u(t) = \begin{cases} 0, & t < 0 \\ 1, & t \geq 0 \end{cases} \tag{S.26}$$

Taking the Laplace transform of Equation (S.26):

$$\overline{\varepsilon} = \frac{\varepsilon_0}{s} \tag{S.27}$$

Substituting Equation (S.27) into Equation (S.25):

$$\overline{\sigma} = \left(E_1 + \frac{E_2 s}{s + \frac{1}{\tau_R}}\right)\overline{\varepsilon} = \left(E_1 + \frac{E_2 s}{s + \frac{1}{\tau_R}}\right)\frac{\varepsilon_0}{s} = \left(\frac{E_1}{s} + \frac{E_2}{s + \frac{1}{\tau_R}}\right)\varepsilon_0 \tag{S.28}$$

Taking the inverse Laplace transform of Equation (S.28), the stress as a function of time in the stress relaxation test using a step function as the loading can be obtained:

$$\sigma(t) = \mathcal{L}^{-1}(\overline{\sigma}) = \left(E_1 + E_2 e^{\frac{-t}{\tau_R}}\right)\varepsilon_0 \tag{S.29}$$

Equation (S.29) is namely Equation (1) in the paper.

On the other hand, in the creep test using a step function as the loading, $\sigma(t)$ is equal to a constant stress value σ_0 times the unit step function:

$$\sigma(t) = \sigma_0 u(t), \text{ where } u(t) = \begin{cases} 0, & t < 0 \\ 1, & t \geq 0 \end{cases} \tag{S.30}$$

Taking the Laplace transform of Equation (S.30):

$$\overline{\sigma} = \frac{\sigma_0}{s} \tag{S.31}$$

Substituting Equation (S.31) into Equation (S.25):

$$\overline{\sigma} = \left(E_1 + \frac{E_2 s}{s + \frac{1}{\tau_R}}\right)\overline{\varepsilon} \Longrightarrow \frac{\sigma_0}{s} = \left(E_1 + \frac{E_2 s}{s + \frac{1}{\tau_R}}\right)\overline{\varepsilon} \Longrightarrow \overline{\varepsilon} = \frac{\sigma_0}{E_1 s + \frac{E_2 s^2}{s + \frac{1}{\tau_R}}} \tag{S.32}$$

Taking the inverse Laplace transform of Equation (S.32), the strain as a function of time in the creep test using a step function as the loading can be obtained:

$$\varepsilon(t) = \frac{\sigma_0}{E_1} - \frac{\sigma_0}{E_1} \cdot \frac{E_1}{E_1 + E_2} \cdot e^{\frac{-E_1}{\tau_R (E_1 + E_2)} t} = \frac{\sigma_0}{E_1} \left(1 - \frac{E_2}{E_1 + E_2} e^{\frac{-t}{\tau_C}} \right) \quad \text{(S.33)}$$

where $\tau_C = \tau_R (E_1 + E_2)/E_1 = \tau_C = \eta (E_1 + E_2)/E_1 E_2$ is the creep time constant. Equation (S.33) is namely Equation (2) in the paper.

4. Derivation of Equations (3) and (4) in the paper

In order to derive the equation form with finite loading rate for analyzing stress relaxation behavior based on the standard linear solid model, the solution for describing the stress-time relationship in the loading process as well as the solution for describing the stress-time relationship in the stress relaxation process in the stress relaxation test are needed. In this section, the derivations of these two solutions are introduced.

Firstly, let's derive the solution for describing the stress-time relationship in the loading process in the stress relaxation test. Let $t = 0$ be the time point at the beginning of the loading process. Let the strain rate in the loading process be r, then the strain at any time in the loading process is $\varepsilon = rt$. Substituting $\varepsilon = rt$ and $\dot{\varepsilon} = d\varepsilon/dt = r$ into the constitutive equation of the standard linear solid model:

$$\sigma + \tau_R\dot{\sigma} = E_1(\varepsilon + \tau_C\dot{\varepsilon}) \implies \sigma + \tau_R\dot{\sigma} = E_1rt + E_1\tau_Cr \tag{S.34}$$

Equation (S.34) is a linear, constant-coefficient, non-homogeneous ordinary differential equation. The initial condition of Equation (S.34) is $\sigma(0) = 0$, since the stress is assumed to be zero at $t = 0$. The general solution of Equation (S.34) is the sum of the homogeneous solution and particular solution.

Consider the homogenous equation of Equation (S.34):

$$\sigma_h + \tau_R\dot{\sigma}_h = 0 \tag{S.35}$$

Let the homogeneous solution be $\sigma_h(t) = Ae^{at}$ and substitute it into Equation (S.35), where A and a are constants:

$$Ae^{at} + \tau_R Aae^{at} = 0 \implies 1 + \tau_R a = 0 \implies a = \frac{-1}{\tau_R} \tag{S.36}$$

Therefore, the homogeneous solution of Equation (S.34) is $\sigma_h(t) = Ae^{\frac{-t}{\tau_R}}$.

Consider the entire equation of Equation (S.34) and let the stress σ be the particular solution σ_p:

$$\sigma_p + \tau_R\dot{\sigma}_p = E_1rt + E_1\tau_Cr \tag{S.37}$$

Let the particular solution be $\sigma_p(t) = Bt + C$ and substitute it into Equation (S.37), where B and C are constants:

$$Bt + C + \tau_R B = E_1rt + E_1\tau_Cr \implies B = E_1r \text{ and } C = E_1r(\tau_C - \tau_R) \tag{S.38}$$

Therefore, the particular solution of Equation (S.34) is $\sigma_p(t) = E_1rt + E_1r(\tau_C - \tau_R)$.

The general solution of Equation (S.34) is the sum of the homogeneous solution and particular solution:

$$\sigma(t) = \sigma_h(t) + \sigma_p(t) = Ae^{\frac{-t}{\tau_R}} + E_1rt + E_1r(\tau_C - \tau_R) \tag{S.39}$$

Substituting the initial condition $\sigma(0) = 0$ into Equation (S.39):

$$\sigma(0) = A + E_1r(\tau_C - \tau_R) = 0 \implies A = -E_1r(\tau_C - \tau_R) \tag{S.40}$$

Substituting Equation (S.40) into Equation (S.39):

$$\sigma_{loading}(t) = E_1rt + E_1r(\tau_C - \tau_R)\left(1 - e^{\frac{-t}{\tau_R}}\right) \tag{S.41}$$

Equation (S.41) is namely the solution for describing the stress-time relationship in the loading process in the stress relaxation test.

Next, let's derive the solution for describing the stress-time relationship in the stress relaxation process in the stress relaxation test. Let $t = 0$ be the time point at the beginning of the stress relaxation process. Let the constant strain in the stress relaxation process be ε_0. Substituting $\varepsilon = \varepsilon_0$ into the constitutive equation of the standard linear solid model:

$$\sigma + \tau_R\dot{\sigma} = E_1(\varepsilon + \tau_C\dot{\varepsilon}) \implies \sigma + \tau_R\dot{\sigma} = E_1\varepsilon_0 \tag{S.42}$$

Equation (S.42) is a linear, constant-coefficient, non-homogeneous ordinary differential equation. The initial condition of Equation (S.42) is $\sigma(0) = \sigma_0$, where σ_0 is the stress at the beginning of the stress relaxation process. The general solution of Equation (S.42) is the sum of the homogeneous solution and particular solution.

Consider the homogenous equation of Equation (S.42):

$$\sigma_h + \tau_R\dot{\sigma}_h = 0 \tag{S.43}$$

Let the homogeneous solution be $\sigma_h(t) = Ae^{at}$ and substitute it into Equation (S.43), where A and a are constants:

$$Ae^{at} + \tau_R Aae^{at} = 0 \implies 1 + \tau_R a = 0 \implies a = \frac{-1}{\tau_R} \tag{S.44}$$

Therefore, the homogeneous solution of Equation (S.42) is $\sigma_h(t) = Ae^{\frac{-t}{\tau_R}}$.

Consider the entire equation of Equation (S.42) and let the stress σ be the particular solution σ_p:

$$\sigma_p + \tau_R\dot{\sigma}_p = E_1\varepsilon_0 \tag{S.45}$$

Let the particular solution be $\sigma_p(t) = B$ and substitute it into Equation (S.45), where B is a constant:

$$B = E_1\varepsilon_0 \tag{S.46}$$

Therefore, the particular solution of Equation (S.42) is $\sigma_p(t) = E_1\varepsilon_0$.

The general solution of Equation (S.42) is the sum of the homogeneous solution and particular solution:

$$\sigma(t) = \sigma_h(t) + \sigma_p(t) = Ae^{\frac{-t}{\tau_R}} + E_1\varepsilon_0 \tag{S.47}$$

Substituting the initial condition $\sigma(0) = \sigma_0$ into Equation (S.47):

$$\sigma(0) = A + E_1\varepsilon_0 = \sigma_0 \implies A = \sigma_0 - E_1\varepsilon_0 \tag{S.48}$$

Substituting Equation (S.48) into Equation (S.47):

$$\sigma_{relaxation}(t) = (\sigma_0 - E_1\varepsilon_0)e^{\frac{-t}{\tau_R}} + E_1\varepsilon_0 \tag{S.49}$$

Equation (S.49) is namely the solution for describing the stress-time relationship in the stress relaxation process in the stress relaxation test.

5. Derivation of Equations (8) and (9) in the paper

In order to derive the equation form with finite loading rate for analyzing creep behavior based on the standard linear solid model, the solution for describing the strain-time relationship in the loading process as well as the solution for describing the strain-time relationship in the creep process in the creep test are needed. In this section, the derivations of these two solutions are introduced.

Firstly, let's derive the solution for describing the strain-time relationship in the loading process in the creep test. Let $t = 0$ be the time point at the beginning of the loading process. Let the stress rate in the loading process be r, then the stress at any time in the loading process is $\sigma = rt$. Substituting $\sigma = rt$ and $\dot{\sigma} = d\sigma/dt = r$ into the constitutive equation of the standard linear solid model:

$$\sigma + \tau_R\dot{\sigma} = E_1(\varepsilon + \tau_C\dot{\varepsilon}) \implies \varepsilon + \tau_C\dot{\varepsilon} = \frac{r}{E_1}t + \frac{r\tau_R}{E_1} \tag{S.50}$$

Equation (S.50) is a linear, constant-coefficient, non-homogeneous ordinary differential equation. The initial condition of Equation (S.50) is $\varepsilon(0) = 0$, since the strain is assumed to be zero at $t = 0$. The general solution of Equation (S.50) is the sum of the homogeneous solution and particular solution.

Consider the homogenous equation of Equation (S.50):

$$\varepsilon_h + \tau_C\dot{\varepsilon}_h = 0 \tag{S.51}$$

Let the homogeneous solution be $\varepsilon_h(t) = Ae^{at}$ and substitute it into Equation (S.51), where A and a are constants:

$$Ae^{at} + \tau_C Aae^{at} = 0 \implies 1 + \tau_C a = 0 \implies a = \frac{-1}{\tau_C} \tag{S.52}$$

Therefore, the homogeneous solution of Equation (S.50) is $\varepsilon_h(t) = Ae^{\frac{-t}{\tau_C}}$.

Consider the entire equation of Equation (S.50) and let the strain ε be the particular solution ε_p:

$$\varepsilon_p + \tau_C\dot{\varepsilon}_p = \frac{r}{E_1}t + \frac{r\tau_R}{E_1} \tag{S.53}$$

Let the particular solution be $\varepsilon_p(t) = Bt + C$ and substitute it into Equation (S.53), where B and C are constants:

$$Bt + C + \tau_C B = \frac{r}{E_1}t + \frac{r\tau_R}{E_1} \implies B = \frac{r}{E_1} \text{ and } C = \frac{r}{E_1}(\tau_R - \tau_C) \tag{S.54}$$

Therefore, the particular solution of Equation (S.50) is $\varepsilon_p(t) = \frac{r}{E_1}t + \frac{r}{E_1}(\tau_R - \tau_C)$.

The general solution of Equation (S.50) is the sum of the homogeneous solution and particular solution:

$$\varepsilon(t) = \varepsilon_h(t) + \varepsilon_p(t) = Ae^{\frac{-t}{\tau_C}} + \frac{r}{E_1}t + \frac{r}{E_1}(\tau_R - \tau_C) \tag{S.55}$$

Substituting the initial condition $\varepsilon(0) = 0$ into Equation (S.55):

$$\varepsilon(0) = A + \frac{r}{E_1}(\tau_R - \tau_C) = 0 \implies A = -\frac{r}{E_1}(\tau_R - \tau_C) \tag{S.56}$$

Substituting Equation (S.56) into Equation (S.55):

$$\varepsilon_{loading}(t) = \frac{r}{E_1}t + \frac{r}{E_1}(\tau_R - \tau_C)\left(1 - e^{\frac{-t}{\tau_C}}\right) \tag{S.57}$$

Equation (S.57) is namely the solution for describing the strain-time relationship in the loading process in the creep test.

Next, let's derive the solution for describing the strain-time relationship in the creep process in the creep test. Let $t = 0$ be the time point at the beginning of the creep process. Let the constant stress in the creep process be σ_0. Substituting $\sigma = \sigma_0$ into the constitutive equation of the standard linear solid model:

$$\sigma + \tau_R\dot{\sigma} = E_1(\varepsilon + \tau_C\dot{\varepsilon}) \implies \varepsilon + \tau_C\dot{\varepsilon} = \frac{\sigma_0}{E_1} \tag{S.58}$$

Equation (S.58) is a linear, constant-coefficient, non-homogeneous ordinary differential equation. The initial condition of Equation (S.58) is $\varepsilon(0) = \varepsilon_0$, where ε_0 is the strain at the beginning of the creep process. The general solution of Equation (S.58) is the sum of the homogeneous solution and particular solution.

Consider the homogenous equation of Equation (S.58):

$$\varepsilon_h + \tau_C\dot{\varepsilon}_h = 0 \tag{S.59}$$

Let the homogeneous solution be $\varepsilon_h(t) = Ae^{at}$ and substitute it into Equation (S.59), where A and a are constants:

$$Ae^{at} + \tau_C Aae^{at} = 0 \implies 1 + \tau_C a = 0 \implies a = \frac{-1}{\tau_C} \tag{S.60}$$

Therefore, the homogeneous solution of Equation (S.58) is $\varepsilon_h(t) = Ae^{\frac{-t}{\tau_C}}$.

Consider the entire equation of Equation (S.58) and let the strain ε be the particular solution ε_p:

$$\varepsilon_p + \tau_C\dot{\varepsilon}_p = \frac{\sigma_0}{E_1} \tag{S.61}$$

Let the particular solution be $\varepsilon_p(t) = B$ and substitute it into Equation (S.61), where B is a constant:

$$B = \frac{\sigma_0}{E_1} \tag{S.62}$$

Therefore, the particular solution of Equation (S.58) is $\varepsilon_p(t) = \frac{\sigma_0}{E_1}$.

The general solution of Equation (S.58) is the sum of the homogeneous solution and particular solution:

$$\varepsilon(t) = \varepsilon_h(t) + \varepsilon_p(t) = Ae^{\frac{-t}{\tau_C}} + \frac{\sigma_0}{E_1} \tag{S.63}$$

Substituting the initial condition $\varepsilon(0) = \varepsilon_0$ into Equation (S.63):

$$\varepsilon(0) = A + \frac{\sigma_0}{E_1} = \varepsilon_0 \implies A = \varepsilon_0 - \frac{\sigma_0}{E_1} \tag{S.64}$$

Substituting Equation (S.64) into Equation (S.63):

$$\varepsilon_{creep}(t) = \left(\varepsilon_0 - \frac{\sigma_0}{E_1}\right) e^{\frac{-t}{\tau_C}} + \frac{\sigma_0}{E_1} \tag{S.65}$$

Equation (S.65) is namely the solution for describing the strain-time relationship in the creep process in the creep test.
