# Supplementary material for: Constitutive Equations for Analyzing Stress Relaxation and Creep of Viscoelastic Materials Based on Standard Linear Solid Model Derived with Finite Loading Rate
Source: Polymers (Basel). 2022 May 23;14(10):2124. doi: 10.3390/polym14102124 (PMC9143375; doi:10.3390/polym14102124)
Supplement: Supplementary file 1 [file polymers-14-02124-s001.zip › Supplementary Material 2.pdf]

Supplementary Material 2

Title of the paper: Constitutive equations for analyzing stress relaxation and creep of viscoelastic materials based on standard linear solid model derived with finite loading rate

Authors: Che-Yu Lin [1,*], Yi-Cheng Chen [1], Chen-Hsin Lin [2] and Ke-Vin Chang [3,4,5,†]
1 Institute of Applied Mechanics, College of Engineering, National Taiwan University, No. 1, Sec. 4, Roo-sevelt Road, Taipei 10617, Taiwan
2 Department of Mechanical Engineering, College of Engineering, National Taiwan University, No. 1, Sec. 4, Roosevelt Road, Taipei 10617, Taiwan
3 Department of Physical Medicine and Rehabilitation and Community and Geriatric Research Center, National Taiwan University Hospital, Bei-Hu Branch, Taipei, Taiwan
4 Department of Physical Medicine and Rehabilitation, National Taiwan University College of Medicine, Taipei, Taiwan
5 Center for Regional Anesthesia and Pain Medicine, Wang-Fang Hospital, Taipei Medical University, Taipei, Taiwan
* Correspondence: cheyu@ntu.edu.tw
† Co-corresponding author.

This supplementary material provides the MATLAB (Mathworks, Natick, Massachusetts, USA) computer programming codes for analyzing stress relaxation behavior by using Equation (7) and creep behavior by using Equation (12). Equations (7) and (12) are the equation forms derived with finite loading rate introduced in this paper for analyzing stress relaxation and creep behaviors respectively. The MATLAB codes can be downloaded from the following link:
https://reurl.cc/Rj0nZG
